# Turkish-German heritage speakers' predictive use of case: webcam-based vs. in-lab eye-tracking

**DOI:** 10.3389/fpsyg.2023.1155585

**Published:** 2023-07-19

**Authors:** Onur Özsoy, Büsra Çiçek, Zeynep Özal, Natalia Gagarina, Irina A. Sekerina

**Affiliations:** ^1^Leibniz Center for General Linguistics (ZAS), Berlin, Germany; ^2^Berlin School of Mind and Brain, Humboldt University of Berlin, Berlin, Germany; ^3^College of Staten Island, New York, NY, United States

**Keywords:** sentence processing, bilingualism, predictive processing, eye-tracking, visual word paradigm, heritage language, Turkish

## Abstract

Recently, Özge et al. have argued that Turkish and German monolingual 4-year-old children can interpret case-marking predictively disregarding word order. Heritage speakers (HSs) acquire a heritage language at home and a majority societal language which usually becomes dominant after school enrollment. Our study directly compares two elicitation modes: in-lab and (remote) webcam-based eye-tracking data collection. We test the extent to which in-lab effects can be replicated in webcam-based eye-tracking using the exact same design. Previous research indicates that Turkish HSs vary more in the comprehension and production of case-marking compared to monolinguals. Data from 49 participants–22 Turkish monolinguals and 27 HSs–were analyzed using a binomial generalized linear mixed-effects regression model. In the Accusative condition, participants looked for the suitable Agent before it is appeared in speech. In the Nominative condition, participants looked for the suitable Patient before it is appeared in speech. HSs were able to use morphosyntactic cues on NP1 to predict the thematic role of NP2. This study supports views in which core grammatical features of languages, such as case, remain robust in HSs, in line with the Interface Hypothesis. We were able to replicate the effect of the predictive use of case in monolinguals using webcam-based eye-tracking, but the replication with heritage speakers was not successful due to variability in data collection contexts. A by-participant analysis of the results revealed individual variation in that there were some speakers who do not use case-marking predictively in the same way as most monolinguals and most HSs do. These findings suggest that the predictive use of case in heritage speakers is influenced by different factors, which may differ across individuals and affect their language abilities. We argue that HSs should be placed on a native-speaker continuum to explain variability in language outcomes.

## 1. Introduction

In languages with flexible word order grammatical case on noun phrases (NPs) is a predictive feature that allows comprehenders to anticipate thematic roles of upcoming referents. Prediction in spoken language comprehension by monolingual adults has been firmly established in the sentence processing research. However, whether children acquiring two L1s and second language (L2) learners can anticipate the thematic roles of NPs based on their grammatical case from the context of the sentence remains open (Pickering and Gambi, 2018; Felser and Arslan, 2019; Karaca et al., 2021b; Kunduz and Montrul, 2022). The inspiration for this line of research comes from the seminal eye-tracking study of Kamide et al. (2003) in which German-speaking adults rapidly used the accusative case on the NP1 (patient *den Hasen*, the_ACC_ rabbit) to predict the NP2 (agent *der Fuchs*, the_NOM_ fox) in the OVS sentences (1) before the latter appeared in spoken input:

(1)       *Den Hasen frisst gleich der Fuchs*. the_ACC_ rabbit eats shortly the_NOM_ fox “The fox will shortly eat the rabbit.”
(2)       *Der Hase frisst gleich den Kohl*. the_NOM_ rabbit eats shortly the_ACC_ cabbage “The rabbit will shortly eat the cabbage.”

In that experiment, participants viewed the pictures of four referents (rabbit, fox, cabbage, tree) as they listened to the spoken sentences (1)–(2) while their eye movements were recorded. Kamide and colleagues found that during the adverb region (shortly) in (1), the listeners looked significantly more to the agent NP (fox) whereas in (2), they looked more to the patient NP (cabbage). Thus, the second referent was anticipated prior to the onset of its name in the spoken input. This shows that speakers can process case-marking cues predictively to incrementally anticipate the upcoming words.

Recently, Özge et al. (2019, 2022) employed the same Visual World design developed by Kamide et al. (2003) and expanded the scope of their investigations to monolingual German- and Turkish-speaking children. Specifically, their research focus in Özge et al. (2019) was on Turkish-speaking children; adult participants as a control group. The study entailed two experimental conditions, with Experiment 1 involving the presentation of sentences in the verb-middle order and Experiment 2, sentences in the verb-final order. The initial finding of the study suggests that children can predictively use the case in their respective languages just like the monolingual adults do, as early as at age of four. The second finding indicates that both monolingual Turkish-speaking adults and children can anticipate the thematic role of the subsequent argument using only NP1 and its case marker, regardless of verb order.

The anticipatory processing of the grammatical case on NPs in sentences with non-canonical OVS word order in bilingual speakers, be it L2 learners or heritage language speakers (HSs), is also debated (Kaan and Grüter, 2021; Soares et al., 2022). As bilinguals often have difficulties with correct interpretation of morphosyntactic information, including the grammatical case (Gor et al., 2019; Ivanova-Sullivan and Sekerina, in press), it is possible that they are less likely to use such information predictively. The findings so far range from no evidence of the prediction (Hopp, 2015; Mitsugi and Macwhinney, 2016) to native-like prediction (Dijkgraaf et al., 2017; Ito et al., 2018b). Moreover, the type of bilingualism, i.e., L2 vs. HSs, that is reflected in differences in proficiency, manner, and timing of acquisition, affects their predictive ability (Karaca et al., 2021b). The influence of demographic and language background factors, such as literacy, age of onset, and language exposure that can affect a speaker's ability to process grammatical cues predictively, is also largely unknown.

Our study builds on Özge et al. (2019) findings that monolingual Turkish-speaking preschool children and adults have predictive abilities in thematic role assignment and test it with bilingual heritage Turkish-German adults. The study has three key purposes: (1) to conceptually replicate Özge and colleagues' hypothesis by extending it to a new population; (2) to compare whether predictive abilities in HSs can be successfully investigated in the Visual World eye-tracking Paradigm (VWP) remotely using a web-based camera on a participant's laptop (Slim and Hartsuiker, 2021; Vos et al., 2021); and (3) explore individual differences in predictive abilities of HSs.

As far as the first purpose is concerned, our study could be thought of as a conceptual replication of Experiment 2 with case-marking cues on NP1 and verb-final order from Özge et al. (2019) because we test the same hypothesis and use experimental design, materials and measures reproduced from Özge and colleagues (Marsden et al., 2018; Grieve, 2021). At this point, the psycholinguistic research community considers testing the generalizability of the prediction hypothesis essential for the theories of psycholinguistics and language acquisition (Huettig and Mani, 2016; DeLong et al., 2017). Because our participants all started as child HSs in families where Turkish was spoken as a home language in Germany, we expected them to be quite similar to monolingual Turkish-speaking children. Later, at school start, they switched to German, the societal language. That is, the school entry is also the start of speaking German mostly in everyday communication (e.g., at school, in the society and public). While Turkish remains a part of everyday communication, it is limited to certain social groups, such as family and friends, who are also Turkish speakers. Many HSs preserve high proficiency, and strong Turkish identity that are characteristic of Turkish HSs residing in Germany (Küppers et al., 2015; Bayram and Wright, 2018). Thus, because the grammatical case acquisition in L1 Turkish is completed way before the age of four (Aksu-Koç and Slobin, 2017), one could expect that Turkish HSs in our sample should anticipate the thematic role of NP2 as soon as they hear NP1, just like monolingual children do.

However, our replication is only conceptual because adult Turkish HSs constitute a new population. In case we find that their predictive abilities differ from those of monolingual children, there may be a number of alternative explanations, including the fact that heritage language grammars can undergo restructuring and/or that HSs can show attrition in their HL with passing time. In heritage language bilingualism, different areas of grammar (Polinsky, 2018), such as syntax and morphology, seem to present difficulties for HSs (Sorace, 2011). This is embedded in an extension of the *Interface Hypothesis* which predicts preservation at the internal interfaces/core grammar (e.g., between morphology and syntax) and problems at the external interfaces (e.g., between syntax and pragmatics; Sorace and Serratrice, 2009). As case-marking is a core grammatical feature of Turkish, we expect to find predictive processing in bilingual heritage Turkish-German speakers too.

Our second key purpose is to examine whether predictive abilities in HSs can be reliably tested without having access to a high-end in-lab expensive equipment, such as stationary eye-trackers (i.e., *EyeLink, Tobii*, and *SMI*). If this is the case, a simple set-up with a webcam-based laptop connected to the Internet will allow us to record eye movements online. It opens up a possibility to vastly expand our current modest efforts to investigate heritage languages that are understudied (or not studied at all). To achieve this purpose, we conducted our eye-tracking experiment twice: first, using a in-lab high-end *Tobii Fusion* 120 Hz eye-tracker (Experiment 3) and then replicating it with *PCIbex*, an open full service platform for online behavioral experiments (Schwarz and Zehr, 2021; Experiments 1 and 2).

Finally, individual variation in demographic and language history background is an important modulator of HSs' ability to process the grammatical case predictively. Parental input, language use, literacy levels, and processing strategies can affect HSs' language processing all the way down to neural signatures in the brain (Soares et al., 2022). Individual variation in HSs is a relatively novel line of research in heritage language bilingualism. Regarding the predictive use of case, it is possible that HSs with higher proficiency and frequent language use of Turkish show this effect while less proficient speakers do not. Therefore, individual variation is part and parcel of the present study as it suggests an alternative (or extension) to the commonly used approach of looking at the participants through the lens of group means.

## 2. Background

### 2.1. Conceptual replication: processing of grammatical case in heritage Turkish

Turkish is a language with very flexible word order even though (S)OV sentences are most common (Göksel and Kerslake, 2004). The present study started as a replication of Experiment 2 by Özge et al. (2019) that compared the predictive abilities of monolingual Turkish-speaking adults and 4-year-old children in verb-final SOV and OSV sentences with overt case-marked subject and direct object. The reason why Özge and colleagues used verb-final sentences was to see whether children could predict the thematic role on the NP2 from just the grammatical case on the NP1, without any additional information from the verb. Indeed, the authors demonstrated that children, like adults, made use of the grammatical case on the NP1 and successfully inferred the thematic role of the NP2. Thus, the case-marking alone, regardless of verb order, could be sufficient for prediction of the upcoming arguments in Turkish. We expect to replicate this finding in our monolingual Turkish-speaking adults using the web-based camera eye-tracking (Experiment 1).

Testing the prediction effect in monolingual Turkish and HSs is important because of the special cross-linguistic contribution that Turkish can make to investigations of predictive language processing. The previous studies of verb-medial languages with the strict SVO word order demonstrated that early grammatical cues from the verb that is located between the NPs produce a strong anticipatory effect on subsequent argument processing (Mani and Huettig, 2012; Gambi et al., 2016). But what happens when some of the cues are late, such as when the verb is in the sentence-final position? There is some evidence that comes from Dutch (Brouwer et al., 2019) and German (Özge et al., 2022), but these languages exhibit less flexible word order, limited case marking, and obligatory overt arguments. Turkish allows us to disentangle the timing effects of the cues that come later, i.e., when the case-markings are at the end of the nouns and the verb is sentence-final.

For Turkish, recent work by Karaca et al. (2022) has been exploring the timing of the cues with HL Turkish-Dutch adults. The preliminary results reveal that HSs process the grammatical case predictively only when lexical and grammatical cues appear early and together, which happens in verb-medial sentences. In contrast, they found no prediction in verb-final sentences. It is possible that it could be due to the difference in the types of cue, in that lexical and semantic cues are stronger whereas grammatical (or morphosyntactic) ones are weaker. In our study, we utilized both types, namely, the early cue in the form of the grammatical case on NP1 and the late lexical cue on the verb in the sentence-final position.

### 2.2. Methodological advancement: comparing in-lab and webcam-based web-based eye-tracking

The few published VWP studies with HSs have employed the stationary high-end in-lab eye-trackers, such as Tobii (Karaca et al., 2022), SMI (Fuchs, 2019), and EyeLink (Sekerina and Sauermann, 2015; Jegerski and Sekerina, 2020; Fuchs, 2022). These eye-tracking studies have reliably measured the timing of cue processing of different phenomena in heritage languages in real-time. However, the progress in studying predictive processing in HLs is slow because stationary in-lab eye-trackers are expensive, require an experienced researcher to control the experiment, and have a long learning curve, which makes them less accessible for researchers in heritage language bilingualism. But every cloud has a silver lining; the recent COVID-19 pandemic has precipitated a potential solution to the prohibitive costs of in-lab eye-tracking, namely, switching to webcam-based eye-tracking with web-based cameras that these days come on most desktop and laptop computers.

The first methodological study assessing the pros and cons of webcam-based eye-tracking in cognitive research was published by Semmelmann and Weigelt (2018). Extending an experimental design used in the in-lab environment to a JavaScript-based eye-tracking algorithm implemented in online environment allowed the authors to compare the accuracy of the two methods in three different tasks: simple fixation, pursuit, and free viewing. Semmelmann and Weigelt, however, reported a greater rate of temporal error when eye movements were collected remotely via participants' web-based cameras on their personal computers because specifications, such as *frames per second (fps)* rates and inter-sampling interval, varied much more than in the stationary in-lab setting.

Recently, the first psycholinguistic VWP experiments conducted remotely using the web-based cameras on participants' computers have appeared. Vos et al. (2021) assessed the predictive processing of verb aspect (simple past vs. progressive) in English-speaking adults. Using WebGazer.js (Papoutsaki, 2015) with an average fps rate of 20.73, the authors replicated their in-lab results obtained with the *SMI Red500* eye-tracker with 64 participants. The looks of 124 online participants to the picture that matched the verb aspect condition were earlier than in the mismatched condition, just like in the in-lab set-up. The authors argued that the web-based cameras were appropriate for investigating fine-grained temporal characteristics of predictive processing despite some minor issues. The latter included (a) the necessity to increase the subject power by at least 30% as 63 online participants did not pass the stringent hardware and calibration control requirements, (b) frequent re-calibration, i.e., every 12 trials, and (c) a 50-ms delay in the onset of the verb aspect effect.

In another recent study, Slim and Hartsuiker (2022) replicated the results of their in-lab VWP experiment (*EyeLink 1000*) of the effect of verb semantics on selection of a referent out of 4 referents presented in quadrants. They used the web-based eye-tracking method (average fps of 18.1) and the module for webcam-based eye-tracking from *PCIbex* (Schwarz and Zehr, 2021). The same issues as in Vos et al. (2021) occurred again, and they were even more substantial. To obtain a sample size of 90 participants, the authors had to (a) recruit 360 people on *Prolific*, (b) were only able to keep participants who obtained a higher calibration score of 50, and (c) found a consistent time lag of 300 ms on average in comparison to the original in-lab timing of the effect of verb semantics.

These studies clearly demonstrate that while web-based eye-tracking delivers good approximation of the location of fixations, it still not sensitive enough to accurately record the timing of eye movements. This is because the typical sampling rate of the consumer-grade web-based cameras, i.e., 24, 30, and 60 fps, is not sufficient to measure rapid eye movements, as opposed to stationary high-end (also known as *infrared*) eye-trackers, which range from 30 to 1,200 Hz (Dalmaijer, 2014; Vos et al., 2022).

Despite the drawbacks of the web-based eye-tracking, its flexibility, low cost, and scalability still present indisputable advantages for research in heritage language bilingualism. Our study is a first rigorous comparison of the (remote) web-based eye-tracking (Experiments 1 and 2) with the stationary in-lab Tobii eye-tracker (Experiment 3) in a VWP study with HSs. We used the same design to ascertain whether the timing of grammatical and lexical cue effects would be comparable in comprehension of SOV and OSV Turkish sentences. The second novelty has to do with the fact that we studied predictive processing with (remote) webcam-based eye-tracking with Turkish HSs. We hope to show what researchers in HL bilingualism need to take into the account when adopting remote web-based eye-tracking to HSs so that it can be established as a widespread, reliable, and accessible research method. Thus, our study addresses an emergent need outlined as necessary for HL bilingualism in the future (Bayram et al., 2021).

### 2.3. Individual differences in predictive abilities

The traditional group mean-based approach to cue predictive processing is expected to confirm that monolingual Turkish-speaking adults can successfully use the grammatical case information on the NP1 to anticipate the thematic role of the NP2. However, because HSs are characterized by large individual variation in their demographic and language history experience in Turkish, averaging their eye-movement patterns may mask the differential predictive abilities of HSs who, we argue, fall into three types—predictors, partial predictors, and non-predictors. We define in detail how we calculated these types in Section 4.4.

The driving force behind these types is what underlies an individual's ability to process sentences predictively or not. Previous literature has suggested several factors that might modulate individual's predictive abilities. The first and most prominent one is proficiency (e.g., Mani and Huettig, 2012; Brouwer et al., 2017; Hopp and Lemmerth, 2018). Heritage speakers are a very heterogeneous group as far as language proficiency is concerned (Wiese et al., 2022). The second factor has to do with typological similarity between the relevant grammatical features in a bilingual's two languages (e.g., Dussias et al., 2013; Foucart et al., 2014). In our study, Turkish and German are similar as both use case marking, which indicates the thematic role of the arguments, i.e., agent (NOM case) or patient (ACC case). However, Turkish is much more consistent in marking the case directly as a suffix on the noun. In contrast, in German, the case-marking system is less transparent. Morphemes that mark case overlap with other grammatical categories such as number and gender. Thus, in the present study, we focused on the category of masculine nouns for NP1 in the items, as this is the grammatical gender in German where accusative and nominative case always unambiguously contrast on the article which is the element in the study designated to allow predictive processing.

Finally, a speaker's cognitive resources is also another indicator of their predictive abilities in real-time processing (Ito et al., 2018a). For example, Huettig and Janse (2016) highlighted the role of working memory in predictive processing of grammatical gender in Dutch participants. Their results showed that faster processing speed and higher working memory capacity facilitated predictive looks. While we have not assessed participants' working memory, we assume that this might be one of the driving factors behind predictive abilities and encourage further work with heritage speakers to explore these aspects.

## 3. Method

The current study consisted of three experiments, i.e., Experiment 1, Experiment 2, and Experiment 3. All of them share the same design but differ either in terms of the group (i.e., monolingual Turkish vs. HSs) or method (i.e., in-lab *Tobii Fusion* 120 Hz vs. webcam-based eye-tracking). Experiment 1 was conducted with monolingual Turkish adults using the web-based camera eye-tracking. Experiments 2 (webcam-based eye-tracking) and 3 (in-lab Tobii eye-tracker) investigated two separate groups of bilingual HL Turkish-German adults with the same linguistic background profile. The community we have worked with is Turkish HSs who live in Berlin, Germany. It is a highly cohesive and vital speech community where Turkish is used on an everyday basis in many informal settings (Özsoy et al., 2022). However, many HSs are the third and fourth generation and they often do not use a strictly monolingual mode when speaking Turkish. More often, they prefer to engage in code-switching and rely on lexical and grammatical borrowings as German is most likely their dominant language as well as the language exclusively used in education (Küppers et al., 2015).

### 3.1. Participants

#### 3.1.1. Experiment 1: monolingual Turkish adults (webcam-based eye-tracking)

Twenty-two monolingual Turkish-speaking participants (59% females, *M*_age_ = 33.5, range 19–63, median_age_ = 25) were recruited from Anadolu University in Eskişehir (Turkey) who participated in the webcam-based eye-tracking experiment. They all were raised monolingually, and their first encounter with another language was in primary school. The data from all 22 participants were included in the analysis.

#### 3.1.2. Experiments 2 and 3: heritage Turkish adults (webcam-based or in-lab Tobii eye-tracking)

Forty Turkish-speaking HSs living in Berlin participated in the study^1^. The first half (*n* = 20, 61% female, *M*_age_ = 24.8, range 18–33, median_age_ = 28) participated in Experiment 2 (webcam-based eye-tracking). The second half (*n* = 20, 66% female, *M*_age_ = 26.3, range 18–35, median_age_ = 31) participated in Experiment 3 (in-lab Tobii eye-tracking). All HSs were recruited from the wider network of acquaintances of the first author and from those who replied to our recruitment flyers.

The participants were all born and raised in Berlin, Germany, and acquired Turkish from birth in their family (age of onset for Turkish was zero). They were the second or third generation of Turkish immigrants, because their (grand)parents moved to Berlin as part of the worker's recruitment agreement between Germany and Turkey in 1961–1973. It is estimated that more than 5% of people in Berlin are Turkish-speaking and in some areas (e.g., Kreuzberg) Turkish can serve as a language of everyday communication in business and shops. This leads to a high level of vitality of the Turkish language among the bilingual Turkish-German speakers. However, only two of our 40 participants have received some level of formal Turkish education at school. The overwhelming majority (i.e., the remaining 38 participants) have received mostly received no education in Turkish, e.g., only 1 year in primary school for 1 hour a week, or none at all. All participants can be assumed to be dominant in German due to its relevance in education, career and overall communication with the mostly German-speaking population (the mean age of onset for German was 6 months, range 0–3 years).

To ensure comparability among the groups, we sampled speakers from the same population who live in similar environments. For example, several of the participants are colleagues at the same workplaces, with certain established language practices. Many of the participants were also recruited directly from the first author's private networks and acquaintances which ensures a certain level of control of the environment. In the recruitment process, participants were required to speak and hear Turkish at home with their families, and they all confirmed that it was the case.

For both experiments, only 27 HSs in total were included in the analysis. In Experiment 2, seven of the 20 webcam-based participants were excluded because of (a) failure to calibrate successfully until the end of the experiment (*n* = 6) and (b) low accuracy score (*n* = 1). In Experiment 3, six of the 20 in-lab participants were removed from the analysis because of (a) technical errors during the recording (*n* = 2), (b) failure to comply with the instructions (*n* = 2), and (c) low accuracy score (below 80%, *n* = 2). In Table 1, we present a summary of common issues with webcam-based eye-tracking and corresponding recommendations. We also compare these issues to our experiences with lab-based high-end eye-tracking.

**Table 1:** Summary of possible issues and corresponding recommendations.

	**Webcam-based eye-tracking**	**Lab-based high-end eye-tracking**
Calibration	Prone to issues because of many varying conditions such as lighting, facial features, webcam-quality • can be immensely improved by careful instructions and in-person or videocall supervision to correct participants posture mistakes or help participant to set up background conditions correctly	Usually very robust and needs minimal instructions that tell the participant to look at the moving dot and not move their head much
Accuracy	Moderate, especially improved, when only participants that calibrate (>50%) well throughout the whole experiment are kept in the sample (for example, Slim and Hartsuiker, 2022 had to exclude 240 out of 330 participants because of insufficient calibration), but importantly it is good enough for quadrant based VWP eye-tracking	High and easy to reach accuracy over 90%
Error-proneness	High (technical usage, hardware, and software variability)	Low, as the experimenter is in the room and can control the devices and surroundings
Lighting conditions	Very important since it is based on visible light, crucial that light is stable and ideally the whole face is well illuminated; avoid distracting light sources from the side or back of the head	Important and needs to be controlled too, but less sensitive since it is based on infrared light
Supervision	Strongly suggested as this improved overall calibration rate immensely (comparing Experiment 1 with supervision and Experiment 2 with only partial supervision); the experimenter can give helpful feedback to help participant calibrate well and keep posture and attention up throughout the whole experiment	Suggested and required to begin the experiment; after a few successful trials, the participant can complete the experiment on their own and the experimenter can retreat to another location in the lab

### 3.2. Design and materials

**Figure 1:**
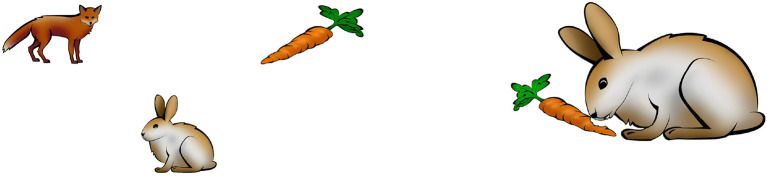
A sample 1st display with the three referents and a sample 2nd display for the picture-sentence-matching for (3).

All three experiments used 20 experimental, 10 filler, and 2 practice items that were adapted from Özge et al. (2019)'s study (see our OSF repository for the complete set of materials). Each item consisted of two visual displays presented in sequence. The first display contained three referent objects (fox, rabbit, carrot) and was projected on the screen (Figure 1). The participant heard a spoken sentence (3) or (4) that described a transitive event that connected the two of the referents (e.g., eating, biting, etc.). After that, the second visual display appeared that depicted the event which either matched or did not match the sentence, e.g., the fox getting ready to eat the rabbit or the rabbit getting ready to eat the carrot (Figure 1). The design was 2-factorial and crossed the independent variable Word Order (SOV vs. OSV)/Case (NOM vs. ACC) as illustrated in (3) and (4); they were manipulated within-participants.

(3)       *Hızlı tavşan şuradaki havuc-u birazdan yiyecek*. fast rabbit.NOM over-there carrot-ACC soon eat “The fast rabbit will soon eat the carrot over there.”
(4)       *Hızlı tavşan-ı şuradaki tilki birazdan yiyecek*. fast rabbit-ACC over-there fox.NOM soon eat “The fox over there will soon eat the fast rabbit.”

The participants' task was picture-matching in choosing whether the depicted event in the second video display matched the sentence that they had heard by pressing the F or J keys to indicate YES or NO answers, respectively. Among the total 30 items (critical and filler), 22 required the YES-answer and 8 required the NO-answer. Their eye movements were recorded during the presentation of both displays, but only the eye-movement patterns during the viewing of the first one (Figure 1) were analyzed as only these are informative regarding predictive processing of case-marking cues.

The spoken sentences were recorded by a monolingually raised female native Turkish speaker^2^ with a focus accent on the verb. The NP1 was followed by 300 ms prosodic break that was judged as natural by a small pilot group of five native speakers. The pictures were color drawings of the referent objects and events taken from Özge et al. (2019) with the permission of the authors. In the experimental items, referents had three possible thematic roles, namely, a topic (i.e., the expressed noun), plausible agent (i.e., instigator of an event), and plausible patient (i.e., the referent that is affected by the instigated event). The referents included animate objects as plausible agents, such as people (e.g., *grandpa, baby*) and animals (e.g., *bear, monkey*), and inanimate objects as plausible patients (e.g., *honey, ice-cream*). There were nine different transitive verbs (e.g., *hit, eat*). Three referents were placed in the visual display (Figure 1) in a triangle, with two in the top row and the third one in the middle of the bottom row. The location of each referent was pseudorandomized, with each thematic role appearing equally in three different locations (upper right, upper left, and lower middle). The video displays and the spoken sentences were combined in the script prepared in the PCIbex. Each video display started with 750 ms of silence and ended with 1,500 ms of silence.

The 10 filler items looked like the experimental items, with three referents in the first video display, and an event in the second display which also required the picture-matching task. Each participant saw the same filler items. The fillers were composed of intransative sentences that started with a complex head-final NP which was preceded by a modifier that was either marked in the GEN case, as in (5), or formed a complex phrase with a non-finite verb, as in (6).

(5)       *Dikkatsiz çocuǧ-un balon-u birazdan patlayacak* careless child-GEN balloon-POSS soon explode “The careless child's balloon will soon explode.”
(6)       *Genç polisin bindiǧi gemi birazdan batacak*. young police-officer enter_NMZ_ boat soon sink “The boat that the young police officer entered will soon sink.”

Four versions of the experiment were created. Experimental items were rotated through the two conditions (Word Order/Case), with five items per condition, in a Latin Square design. Participants in each experiment were randomly assigned to one of the four versions and responded to 20 items in total, including 10 filler items.

In addition to this experimental task, there was also a participant background questionnaire. The online version was directly implemented in PCIbex and was the second to last display that participants saw. The final display was a thank you screen with contact information of the experimenter. The offline version of the questionnaire was handed out in paper form. It contained six questions about the participants' gender, place of birth, place of residence, age of onset for both their languages and cumulative years in formal education (starting from primary school onward).

### 3.3. Procedure

#### 3.3.1. Experiments 1 and 2: webcam-based eye-tracking

All experiments as well as the procedures were approved by the ethics committee of the German Linguistic Society (Deutsche Gesellschaft für Sprachwissenschaft) with the votum #2022-02-220202. The study was implemented on the *PennController for Internet-Based Experiments* (PCIbex) platform (Schwarz and Zehr, 2021). PCIbex uses the WebGazer.js eye-tracking library which can track participants eye movements using standard computer webcams (Papoutsaki et al., 2016). The script of the experiment was programmed using PCIbex's own simple language in a main JavaScript document. Modifications in the script were made offline and the updated script was then uploaded into the respective section of the PCIbex project overwriting the previous version. This ensured that all changes were saved and is recommended since there is a bug in PCIbex's autosave function. We uploaded all our experimental image and audio files directly into PCIbex “Ressources” section. The detailed documentation at https://doc.pcibex.net/ outlines how different elements and whole experiments can be set up in PCIbex. The webcam video is converted into eye-tracking data in the participants browser. The eye-tracking data run through a PHP script that renders them into a standard data spreadsheet. This script needs to be stored externally and it also stored the resulting eye-tracking data there which is why it requires write-access on the server. In line with the European General Data Protection Regulation, we used our own server at the Humboldt-Universität zu Berlin for this purpose. Once the script was ready, PCIbex generated a web link that we provided to the participants (see the demo of the full script of the experiment). A 12.66” *Dell Inspiron* 7,400 laptop with a 30-fps web camera and the Internet connection was used. During Experiment 1 (monolingual Turkish speakers), the laptop was housed in a soundproof eye-tracking laboratory of Anadolu University in Eskisehir (Turkey). The appropriate lighting, noise-proof environment, and reliable Internet connection in this lab were ideal for eye-tracking experiments. All 22 monolingual participants were tested on this laptop in the same location, with the experimenter present. Experiment 2 (Turkish HSs) was conducted in the field in Berlin, and the conditions varied much more due to changing testing environments. Twenty HSs participated at their homes or their workplaces in a quiet location. Among them, four HSs completed the experiment on their own personal computers and 16 HSs completed the experiment from the experimenter's Dell Inspiron 7,400 that was used with the monolingual speakers in Experiment 1. Variability in hardware and field conditions explains why the data from only 13 HSs were usable and included in the analysis.

At the beginning of the experiment, participants read the description of the experiment, electronically signed the consent form, and filled out the online demographic and language background questionnaire. Then they were asked to self-calibrate by following the instruction on the screen. Calibration was better when the experimenter was present and could assist participants by adjusting the laptop screen to the appropriate angle and optimizing the lighting conditions and background colors. Still, some participants failed to calibrate either due to one of the aforementioned variables or due to other factors such as facial features or webcam quality. Following successful calibration, participants started the experiment with two practice trials followed by 20 experimental trials interspersed with 10 fillers. It took participants on average 10 min to complete the task itself.

#### 3.3.2. Experiment 3: in-lab stationary Tobii eye-tracking

The experiment was conducted in the psycholinguistics laboratory of the Leibniz-ZAS in Berlin (Germany). Individual 30-min appointments were scheduled with each participant based on their availability. The participant was seated in front of the stimuli computer of the high-end stationary *Tobii Pro Fusion* 120 Hz eye-tracker. Calibration was controlled by the *Tobii Pro Lab* software and was validated by the experimenter. When participants looked away from the stimuli computer (e.g., toward the experimenter when asking questions), re-calibration was performed. Just like in Experiments 1 and 2, following the calibration, participants started the experiment with two practice trials followed by 20 experimental trials interspersed with 10 fillers. Participants completed the task itself in around 5 min which is faster than in Experiments 1 and 2 because in-lab stationary eye-tracking required fewer recalibrations and adjustments of the experimental set-up.

### 3.4. Data analysis

A data analysis plan and accompanying predictions were registered in advance of carrying out this study on the AsPredicted web site: https://aspredicted.org/8B7_565. In addition to the registered analysis, we also conducted a divergence point analysis by closely following the procedure and script described by Stone et al. (2021). The eye-movement data were preprocessed and analyzed using R (R Core Team, 2022). We used the following packages: *tidyverse* (Wickham et al., 2019), *lme4* Bates et al. (2015), *boot* (Davison and Hinkley, 1997), *mgcv* (Wood, 2003), and *polycor* (Fox, 2022).

## 4. Results

### 4.1. Accuracy in the picture-matching task

Both groups of participants performed the picture-matching task at ceiling: on average, the 22 monolingual speakers' accuracy was 94% (range 80–100%), the 27 HSs' accuracy was 95% (range 90–100%). This implies that the participants were highly attentive during the experiment.

### 4.2. Analysis of eye movements 1: agent Preference

We start by presenting the analysis of fixation data using a binary dependent variable called *Agent Preference* that we computed following Özge et al. (2019) and Özge et al. (2022). It included only the looks to the plausible Agent or Patient of an item. All other looks were excluded from this variable as they are not relevant for the prediction effect under investigation. Different eye-movement patterns in Agent Preference allow us to directly compare looks in the two Case conditions, ACC and NOM, to test whether in the ACC condition, there was a statistically significant increase in looks to the plausible Agent during the NP2 (2,300–2,600 ms) but before it ends. Such an increase would indicate predictive processing based on the ACC case marker on the NP1 in the OSV sentences.

**Figure 2:**
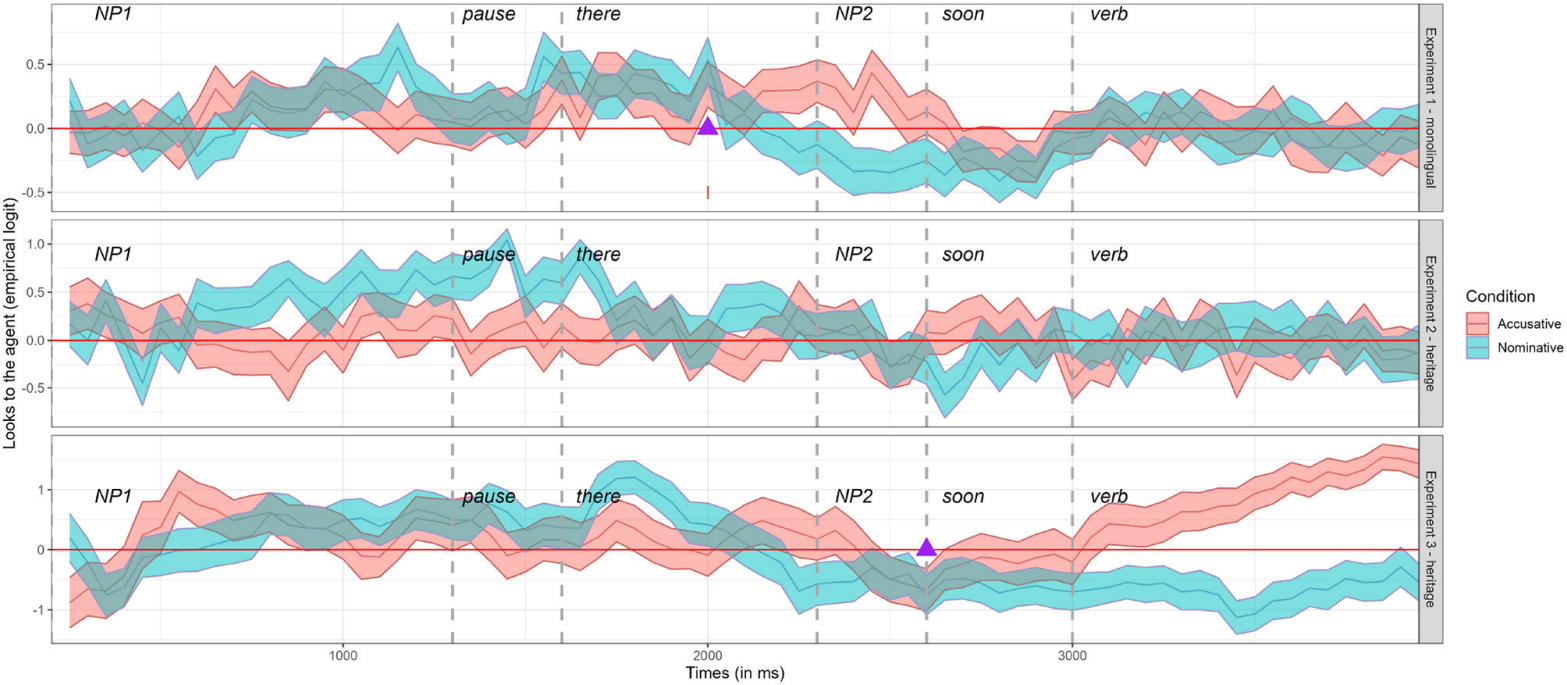
Mean gaze patterns of agent preference in each time window for monolingual and heritage speakers with a webcam-based or lab-based eye-tracker are reflected by the lines. Error bars in blue and red indicate the standard error of the mean. Values above zero indicate preference to look at potential agent, and values below zero indicate a preference to look at the potential patient. Blue (nominative) and red (accusative) also represent the two conditions of the experiment. The purple triangle shows the estimated divergence point for Experiments 1 and 3.

The Agent preference results are shown in Figure 2. The top panel represents Experiment 1 (monolingual speakers, the webcam-based eye-tracking), the middle panel is Experiment 2 (HSs, webcam-based eye-tracking), and the bottom panel, Experiment 3 (HSs, in-lab Tobii eye-tracking). The region of interest for the effect of predictive case marking begins after the 300-ms prosodic break that follows the end of NP1 (1,600 ms). The region of interest ends as soon as the NP2 is encountered in speech (2,600 ms). Any looks following the region of interest are no longer purely predictive because they are based on lexical or prosodic information from the NP2.

In the top panel for Experiment 1, we see that monolingual speakers show an effect of Agent preference around 2,000 ms. After the end of NP2 this effect fades and looks to the Agent and the Patient become roughly equal again. The middle panel for Experiment 2 shows no clear pattern of Agent preference. Throughout most of the time windows, the proportion of looks to the Agent and the Patient do not diverge in a meaningful way. This could partly be due to the low resolution and variable experimental set-up in the webcam-based eye-tracking with heritage speakers, and partly due to a missing predictive processing effect. Experiment 3 (HSs, in-lab eye-tracking, bottom panel, Figure 2) yielded the clearest Agent preference in the ACC condition due to the high resolution and better quality of the in-lab stationary eye-tracker. The HSs' looks to the Agent (above the zero line) and the Patient (below the zero line) clearly increase in the ACC condition OSV (red line for ACC, blue for NOM) after 300 ms from the onset of the NP2, which happens before the end of the NP2 (2,600 ms).

### 4.3. Analysis of eye movements 2: generalized linear mixed model

The first goal of this study was a conceptual replication of Özge et al. (2019). We analyzed eye movements using binomial generalized linear mixed effects regression models (GLMMs). We limited this analysis to the region of interest between the onset of the NP1 and the offset of the NP2 where predictive looks were expected to occur. Because looks to the Agent and the Patient were equal in the critical region in Experiment 2 (HSs, webcam-based eye-tracking) and this clearly indicating that there is no effect, we only compared the results of Experiment 1 (monolingual webcam-based eye-tracking) and Experiment 3 (HSs, in-lab eye-tracking) to estimate meaningful effects in two regression models. Table 2 presents the first, baseline, set of models with the exact same structure (1):

**Table 2:** Experiment 1 (monolinguals, webcam-based eye-tracking) and Experiment 3 (HSs, in-lab eye-tracking): agent preference in the predictive region of interest.

	**Dependent variable**	
	**Agent preference**	**(AgentPrefScore)**
	**Monolinguals**	**HSs**
Condition_AvN	−0.583	0.190
	(0.382)	(0.813)
No. of observations	3,167	2,802

The binary variable Condition_AvN encodes the Accusative and Nominative conditions in the experiment.

(1) *glmer(data=dat, AgentPrefScore Condition_AvN 1 + [1*|*Participant) + (1*|*Item), family=binomial, control=glmerControl(optimizer=“bobyqa”)]*

There was no significant effect in the Agent preference looks between the two groups of participants in this baseline model.

In the second set of models, we incorporated Time as a variable in the form of 100-ms bins that were used to split the region of interest between the onset of the NP1 and the offset of NP2 (This region spanning 2,000–2,600 ms is set out in lavender in Table 3). Table 3 presents the results for these omnibus models. We added TimeWindows as an independent variable and tested the interaction between TimeWindows and Condition on Agent preference (2):

**Table 3:** Two omnibus regression models, one for monolinguals and one for heritage speakers, in a table with the interaction of condition and time (in 100 ms bins).

	**Dependent variable**	
	**AgentPrefScore**	
	**Monolinguals**	**Heritage**
Condition_AvN	−0.151	0.644
	(0.475)	(0.595)
Time2100	−0.103	0.299
	(0.263)	(0.286)
Time2200	−0.030	0.188
	(0.259)	(0.292)
Time2300	0.064	0.208
	(0.259)	(0.306)
Time2400	−0.121	−0.318
	(0.259)	(0.300)
Time2500	−0.280	**−1.014** ^***^
	(0.257)	(0.289)
Time2600	**−0.553** ^**^	**−0.876** ^***^
	(0.251)	(0.294)
Time2700	**−1.002** ^***^	−0.443
	(0.246)	(0.291)
Time2800	**−1.035** ^***^	**−0.607** ^**^
	(0.247)	(0.283)
Time2900	**−0.952** ^***^	**−0.545** ^*^
	(0.246)	(0.289)
Condition_AvN:Time2100	−0.262	**−0.776** ^*^
	(0.380)	(0.398)
Condition_AvN:Time2200	**−0.673** ^*^	**−1.491** ^***^
	(0.374)	(0.419)
Condition_AvN:Time2300	**−0.784** ^**^	**−1.769** ^***^
	(0.380)	(0.424)
Condition_AvN:Time2400	**−0.763** ^**^	**−1.204** ^***^
	(0.375)	(0.414)
Condition_AvN:Time2500	**−0.628** ^*^	−0.347
	(0.367)	(0.400)
Condition_AvN:Time2600	−0.357	**−0.674** ^*^
	(0.367)	(0.404)
Condition_AvN:Time2700	0.120	**−0.883** ^**^
	(0.362)	(0.398)
Condition_AvN:Time2800	0.093	**−1.015** ^***^
	(0.363)	(0.394)
Condition_AvN:Time2900	0.077	**−1.024** ^***^
	(0.359)	(0.396)
Observations	3,167	2,802

^*^p < 0.1; ^**^p < 0.05; ^***^p < 0.01. Bold values indicate significant effects.

(2) glmer[data=dat, AgentPrefScore Condition_AvN * TimeWindows 1 + [1|*Participant) + (1*|*Item), family=binomial, control=glmerControl(optimizer=“bobyqa”)]*

The results showed that there were several significant relationships between TimeWindows and Agent preference between Experiment 1 (monolingual, webcam-based eye-tracking) and Experiment 3 (HSs, in-lab eye-tracking) (marked in boldface in Table 3). In Experiment 1, there were significant negative relationships between four Time bins (i.e., Time2600, Time2700, Time2800, and Time2900) and Agent preference, such that 1-unit increase in the Time bin windows was associated with a 0.553-, 1.002-, 1.035-, and 0.952-unit decrease in Agent preference, respectively (all *p* < 0.01). Significant interactions between Condition and TimeWindows were found at Time2200, Time2300, Time2400, and Time2500, with a negative relationship with Agent Preference corresponding to decreases ranging between 0.628 and 0.784 units.

In Experiment 3 (HSs, in-lab eye-tracking), there were also significant negative relationships between almost the same four Time bins (i.e., Time2500, Time2600, Time2800, and Time2900) and Agent preference, such that 1-unit increase in the Time bins was associated with a 1.014-, 0.876-, 0.607-, and 0.545-unit decrease in Agent preference, respectively (all *ps* < 0.05). Also, significant interactions between Condition and TimeWindows were found at Time2100, Time2200, Time2300, Time2400, that continued at Time2600, Time2700, Time2800, and Time2900, with a negative relationship to Agent preference corresponding to decreases ranging between 0.674 and 1.769 units.

As far as our first goal was concerned, Experiment 1 replicated Özge et al. (2019)'s findings where monolingual Turkish-speaking adults and children as young as 4 years of age made use of the grammatical case on the NP1 and successfully inferred the thematic role of the NP2. We also extended these findings to adult Turkish HSs (our first goal) in Experiment 3. In heritage speakers and monolinguals (Experiment 1 and 3), the significant interactions at the Time2100 and Time2200 windows point to the Case effect before the onset of the NP2 at 2300 ms. Thus, the case-marking alone, regardless of verb position, was sufficient for prediction of the upcoming arguments in monolingual Turkish adults and children, and in HSs.

For our second goal, webcam vs. in-lab replication, have two observations. Firstly, our results from Experiment 1 represent a conceptual replication of Özge et al. (2019), although the low resolution webcam-eye-tracking data shows a smaller effect of predictive use of case. Concerning the differences for HSs between Experiments 2 and 3, we do not find any significant effects in the HSs webcam-based data. The reason most likely lies in the small sample size and the variable experimental set-up for Experiment 2. When the experimental set-up is more stable as in Experiment 1, webcam-based eye-tracking is more feasible and can replicate previous findings.

To establish the precise point where the looks to the Agent diverge from the looks to the Patient in the OSV condition compared to those in the SOV condition in the two groups of participants, we followed Stone et al. (2021) and conducted divergence point estimation using corrected and uncorrected multiple comparisons. Surprisingly, the results in this analysis differed from the GLMM analysis. In this case, the group in Experiment 1, i.e., monolinguals showed a significant divergence point that indicated predictive use of case-marking cues. The group in Experiment 3, i.e., heritage speakers, showed a divergence point that lied behind the prediction region. We present this analysis in detail in Appendix and discuss differences in methodologies.

### 4.4. Individual variation: predictor categories

**Figure A1:**
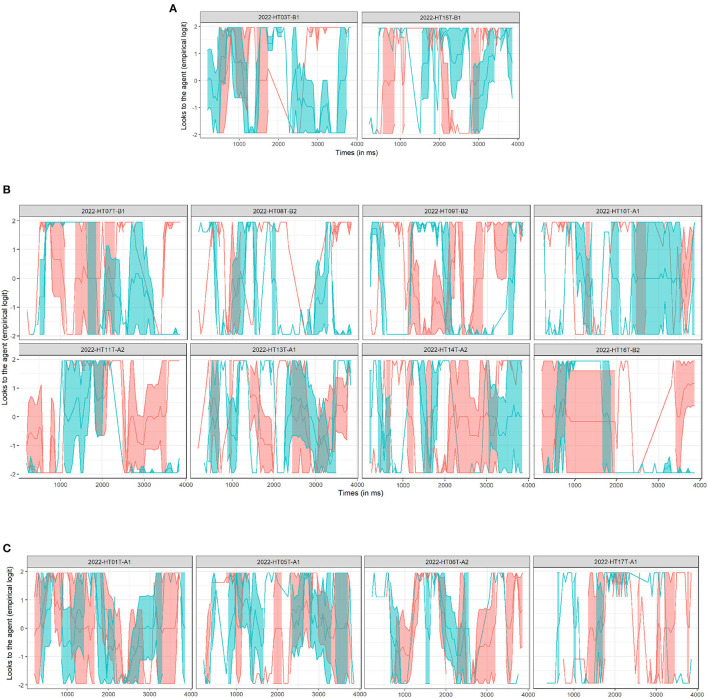
The three subfigures represent individual speakers gaze patterns over all trials. **(A)** This figure presents data from participants who were classified as predictors meaning that they show a consistent predictive use of case before the start of the second NP. **(B)** This figure presents data from participants who were classified as late-predictors meaning that they show a consistent predictive use of case before the full reveal of the second NP. **(C)** This figure presents data from participants who were classified as non-predictors meaning that they show no consistent predictive use of case before the full reveal of the second NP.

Our third goal was to investigate individual ability of HSs to process the grammatical case in the OSV sentences predictively. In line with previous psycholinguisitic research (Hopp and Lemmerth, 2018; Brouwer et al., 2019; Karaca et al., 2021a), we hypothesized that HSs' participant background variables might have an effect on whether they can engage in predictive processing. Based on individual speakers growth-curve figures, we found three types of predictive processing behavior–i.e., predictor, partial predictor, and non-predictor—that are reflected in the Figure A1.

We also found a different and possibly more reliable way, to characterize individual speakers into predictors vs. non-predictors. Since the 2,200–2,300 ms time window is crucial for any predictive looks before the onset of the second NP at 2,300 ms, we focused on this time window. We then calculated the mean AgentPreferenceScore, i.e., whether the participant looked more to the potential agent or the possible patient, in this time window and this was limited to the Accusative condition as it included the first case-marked NP which could serve as a cue. If a person had a score above 0.5, we classified them as *predictors* since they looked to the agent were above chance. If a person had a score below 0.5, this indicated that they were not looking at the agent predictively, so we classified them as *non-predictors*. Table 4 shows the results of this classification. Most of the participants who we classified as predictors are actually well above chance ranging from 75 to 100% which is a clear indicator that they process case predictively. In the monolingual as well as the heritage samples, we see participants who are classified as non-predictors and predictors. Compared to the group size, the proportion of non-predictors in the heritage speaker group is higher than in the monolingual group. However, in both groups there are also more predictors than non-predictors. Additionally, there is a limited number of partial predictors who seem to use case predictively at a chance level of 50% according to this threefold classification. This simple classification sheds light at structured individual variation that must be based on speakers' individual background factors. Future studies should carefully investigate especially sociolinguistic background variables (e.g., proficiency, language exposure) to be able to determine what drives predictive abilities in speakers.

**Table 4:** A categorization of monolingual and heritage speakers into *predictors, partial predictors*, and *non-predictors*.

	**Participant**	**Condition**	**Group**	**Mean**	**Type**
1	1660552776	Accusative	MSwebcam	0.00	**Non-predictor**
2	1660555491	Accusative	MSwebcam	0.57	Predictor
3	1660562087	Accusative	MSwebcam	0.71	Predictor
4	1660565335	Accusative	MSwebcam	0.50	Partial-predictor
5	1660566543	Accusative	MSwebcam	0.25	**Non-predictor**
6	1660568100	Accusative	MSwebcam	0.57	Predictor
7	1660570668	Accusative	MSwebcam	0.50	Partial-predictor
8	1660571549	Accusative	MSwebcam	0.69	Predictor
9	1660572917	Accusative	MSwebcam	0.80	Predictor
10	1660573937	Accusative	MSwebcam	0.73	Predictor
11	1660579497	Accusative	MSwebcam	0.00	**Non-predictor**
12	1660651886	Accusative	MSwebcam	0.50	Partial-predictor
13	1660652919	Accusative	MSwebcam	0.50	Partial-predictor
14	1660653654	Accusative	MSwebcam	0.83	Predictor
15	1660655013	Accusative	MSwebcam	0.71	Predictor
16	1660655834	Accusative	MSwebcam	1.00	Predictor
17	1660656355	Accusative	MSwebcam	0.71	Predictor
18	1660732813	Accusative	MSwebcam	1.00	Predictor
19	1660734167	Accusative	MSwebcam	0.67	Predictor
20	1660826578	Accusative	MSwebcam	0.25	**Non-predictor**
21	1660830489	Accusative	MSwebcam	0.73	Predictor
22	1660831171	Accusative	MSwebcam	0.57	Predictor
23	2022-HT01T-A1	Accusative	HSlab	0.31	**Non-predictor**
24	2022-HT05T-A1	Accusative	HSlab	0.00	**Non-predictor**
25	2022-HT06T-A2	Accusative	HSlab	0.00	**Non-predictor**
26	2022-HT07T-B1	Accusative	HSlab	0.50	Partial-predictor
27	2022-HT08T-B2	Accusative	HSlab	1.00	Predictor
28	2022-HT09T-B2	Accusative	HSlab	0.79	Predictor
29	2022-HT10T-A1	Accusative	HSlab	1.00	Predictor
30	2022-HT11T-A2	Accusative	HSlab	1.00	Predictor
31	2022-HT13T-A1	Accusative	HSlab	1.00	Predictor
32	2022-HT14T-A2	Accusative	HSlab	0.60	Predictor
33	2022-HT15T-B1	Accusative	HSlab	0.20	**Non-predictor**
34	2022-HT16T-B2	Accusative	HSlab	1.00	Predictor
35	2022-HT17T-A1	Accusative	HSlab	1.00	Predictor
36	1646749940	Accusative	HSwebcam	1.00	Predictor
37	1647091947	Accusative	HSwebcam	0.75	Predictor
38	1647605908	Accusative	HSwebcam	1.00	Predictor
39	1647696601	Accusative	HSwebcam	1.00	Predictor
40	1647697953	Accusative	HSwebcam	0.00	**Non-predictor**
41	1649074797	Accusative	HSwebcam	0.67	Predictor
42	1651350251	Accusative	HSwebcam	0.33	**Non-predictor**
43	1651351209	Accusative	HSwebcam	0.83	Predictor
44	1651416084	Accusative	HSwebcam	0.33	**Non-predictor**
45	1652102291	Accusative	HSwebcam	0.00	**Non-predictor**
46	1652103834	Accusative	HSwebcam	0.67	Predictor
47	1652787358	Accusative	HSwebcam	0.43	**Non-predictor**
48	1653463987	Accusative	HSwebcam	0.25	**Non-predictor**

## 5. Discussion

Our findings in multiple groups and using lab-based as well as webcam-based eye-tracking reveal several new insights regarding the predictive processing of case in Turkish heritage and monolingual speakers. Overall, we replicate Özge et al. (2019)'s findings for monolinguals using the webcam-based eye-tracking method. Our monolingual group was able to process case predictively before the onset of NP2. Our analysis located the divergence point at 2,000 ms which is 300 ms before the crucial onset and indicates the use of predictive processing. In contrast, for the heritage speaker group in the lab, our analysis located this divergence point at 2,600 ms which is 300 ms after the onset of NP2. Hence, we observe that heritage speakers, on a group-level, do not process case predictively.

However, our aim was to look further into this issue with more detailed analyses. The first step toward this came from conceptually replicating the same GLMM analysis as in Özge et al. (2019). We observed interactions between Condition and the predictive Time Windows of 2,100–2,300 ms in both groups. Contrary to the results from the divergence point estimates above, this indicates that there is predictive use of case in both groups and not just the monolingual group. This divergence in results points to the relevance of using appropriate methods when analyzing such large data sets across different groups (Vasishth, 2022). Relatedly, recently discussions about accepting uncertainty in experimental studies have emerged (e.g., Vasishth and Gelman, 2021) which we acknowledge by listing some limitations below. It also indicates that group-level analyses might not be the best option for such effects that may be guided by individual speakers abilities and backgrounds which is in line with recent proposals in heritage language research to consider the speaker more (Luk, 2022; Rothman et al., 2022).

To understand this individual variation better, we explored two new ways to categorize the predictive processing of case on a speaker-level. The first one is based on growth curves and the divergence between the conditions at three different time windows of interest as seen in the Figure A1. Based on this, we classified the use of predictive processing in heritage speakers lab-based data. The classification revealed that the majority of participants showed some predictive processing of case before the onset of NP2. However, this method was vague and harder to quantify. Therefore, we used our knowledge about the interaction effects from the GLMM which informed us that there were predictive processing effects in the crucial last 2,200–2,300 ms time window before the onset of the second NP. Our analysis of AgentPreference looks on a speaker-level again showed us that most speakers in the heritage as well as the monolingual group used case predictively.

These detailed analyses allow us to add to group-level analyses to better understand how patterns of predictive processing of case are distributed among different speaker groups. Proportionally, more monolingual than heritage speakers process case predictively. The underlying factors of these results are most likely guided by individual cognitive capacities and other (linguistic) background variables. These expectations are based on previous literature that has shown an effect of these factors on predictive abilities such as Karaca et al. (2021a) who have shown effects of processing speed and language proficiency. To explore these factors further will be an important next step in predictive processing research in the future.

### 5.1. Do heritage speakers process grammatical case predictively?

The present study aimed to investigate the use of predictive case-marking in Turkish-German heritage speakers (HSs) using both in-lab and webcam-based eye-tracking methods, and to explore individual variation among HSs in their use of this grammatical feature. Our results showed that HSs were able to use morphosyntactic cues to predict the thematic role of NP2, supporting the idea that core grammatical features of languages remain robust in HSs. However, a by-participant analysis revealed individual variation in the use of predictive case-marking, with some speakers showing patterns similar to monolinguals and others showing divergent behavior.

These findings have several implications for our understanding of heritage language acquisition and processing. First, they support the view that HSs should be placed on a native-speaker continuum rather than being treated as a homogenous group. Previous research has demonstrated that HSs can show a range of proficiency levels in their heritage language, with some exhibiting near-native abilities and others exhibiting more limited proficiency (e.g., Bayram et al., 2021). Our results suggest that this individual variation may extend to the use of predictive case-marking, with some HSs exhibiting patterns similar to monolinguals and others showing differences. This highlights the importance of considering individual differences when studying heritage language acquisition and processing.

Our results support the idea that core grammatical features of languages, such as case-marking, remain robust in HSs. This is in line with the Interface Hypothesis, which proposes that certain aspects of grammar, such as argument structure and the expression of agreement, are resistant to interference and erosion in bilingual speakers (e.g., Sorace, 2011). This suggests that heritage speakers may have a strong foundation in their heritage language, even if they are not fully proficient in it.

### 5.2. Is it possible to replicate in-lab findings with web-based eye-tracking?

Our study adds to the small but growing body of research on the use of webcam-based eye-tracking methods in psycholinguistic research. Webcam-based eye-tracking allows researchers to collect data from participants in their own naturalistic environments, rather than requiring them to come to a laboratory setting. It allowed us to recruit some heritage speakers, who may not have easy access to a laboratory or may be geographically dispersed. Additionally, we were able to collect data in Türkiye without requiring expensive high-end eye-tracking equipment. Our results from the monolingual group showed that the in-lab and webcam-based eye-tracking data were largely consistent, indicating that webcam-based eye-tracking may also be a viable method for studying heritage language processing. However, further research is needed to fully understand the potential effects, benefits and challenges of webcam-based data collection in psycholinguistic studies. Many of the aspects that have also been found to be crucial in the two other psycholinguistic webcam-based eye-tracking studies by Slim and Hartsuiker (2022) and Vos et al. (2022) turned out to be relevant for the present study too. In particular, researchers need to be aware of the critical conditions that affect data quality when applying webcam-based eye-tracking. To get the most out of this technology, an ideal lab-like setup with good lighting conditions, an undisturbed environment and a stable/consistent internet connection are minimum requirements. Additionally, participants should be closely guided any possibly monitored throughout the process of calibration and later stages of completing the experiment.

Furthermore, Steffan et al. (2023) in a much more large-scaled study have shown that the sampling rate varies between participants due to different hardware conditions. Partially, these differences also stem from the different underlying techniques between webcam- and high-end lab eye-tracking. The former predicts the gaze based on the whole face focusing on the eye using visible light, and the latter tracks the movement of the eye focusing on the pupil using infrared light (Papoutsaki et al., 2016).

### 5.3. Can we better account for individual differences in eye movements?

In an attempt to move the field of heritage language research forward, much recent and some earlier discussion arose about moving away from dichotomous approaches to heritage grammars such as monolingual vs heritage, native vs nonnative, complete vs incomplete, baseline vs. divergence (Cabo and Rothman, 2012; Putnam and Sánchez, 2013; Rothman et al., 2022; Wiese et al., 2022). In line with this current stream, we observe that it comes short to just classify the monolingual group in Experiment 1 as showing a predictive effect, and the heritage groups in Experiments 2 and 3 as not showing it. We explored more nuanced ways to classify not just between groups but rather between speakers. This allowed us to see that we find different types of predictors in all our experimental groups: non-predictors, partial predictors and predictors. More extensive by-participant variables such as language proficiency or working memory scores would provide a better testing ground to be able to determine what influences individual's abilities to predictively process case, and hence be categorized into one of the three predictor types.

Future research can pick up this idea of more nuanced classifications that can also move in a gradient direction. For example, Kutlu et al. (2022) in this same special issue, demonstrates how a traditionally discretely categorized phenomenon such as speech perception can become more gradient to address bilingual speakers speech using possible more adequate methods and tools. In a similar way, we find that empiricially more interesting patterns emerge when we move beyond the dichotomy of mono- vs. bilinguals and instead address the gradiency within these groups. We can now ask what common background variables characters mono- and bilingual speakers who use case predictively to different degrees. Having and including more extensive information about speakers like known parameters such as working memory, proficiency and literacy (Hopp, 2015; Huettig and Janse, 2016; Hopp and Lemmerth, 2018) will help us to understand and explain in different ways how bilingual sentence processing works in the mind.

### 5.4. Limitations

There are several limitations to consider in the present study. First, four participants completed the experiment using their own PCs in different locations, which may have introduced variations in monitor settings that could have affected the results of the webcam-based experiments. Additionally, internet connection quality may have varied across the different locations where participants completed the webcam-based experiments, which could also have influenced the results.

Second, we were not able to collect as much data as we had originally planned, and some data had to be eliminated due to technical issues or participant errors. This may have limited the power of our statistical analyses and could have introduced bias in the results.

Third, we did not collect in-lab eye-tracking data from monolinguals in this study, which means that it is not possible to directly compare the performance of monolinguals and HSs in the same experimental conditions. This is an important direction for future research, as it would provide more insight into the relationship between heritage language proficiency and the use of predictive case-marking.

Finally, it is important to note that our sample was relatively small and may not be representative of all Turkish-German HSs who represent and extremely diverse group (Küppers et al., 2015). Keeping in mind previous literature that has shown that webcam-based eye-tracking requires much larger sample sizes than in-lab eye-tracking, our study should be viewed as a starting point in using this method whose capacity to generalize is limited at this point. Further research with larger and more diverse samples would be needed to confirm and extend the findings of the present study. Alternatively, instead of recruiting different groups of people for each experiment, split-half procedures could have been used on each group (i.e., HSs and monolinguals) to minimize the individual differences in the HSs' profiles and to keep the computer settings constant for all participants. By increasing the number of items, applying split-half producers could be an option for future studies with more accessible populations such as “monolingual” German speakers. Because heritage speakers of Turkish are relatively difficult to recruit due to a smaller community size among other factors, and because webcam-based eye-tracking requires much larger sample sizes to be exactly comparable to lab-based eye-tracking (Slim and Hartsuiker, 2022), this procedure was not feasible for this study.

## 6. Final remarks

In conclusion, the present study provides new insights into the use of predictive case-marking in Turkish-German HSs and the importance of considering individual differences in the study of heritage language acquisition and processing. Our results support the idea that core grammatical features of languages remain robust in HSs and suggest that webcam-based eye-tracking may be a useful method for studying heritage language processing. Future research could further explore the relationship between proficiency in the heritage language and the use of predictive case-marking in HSs, as well as the potential effects of webcam-based data collection on the results of eye-tracking studies.

## Data availability statement

The datasets presented in this study can be found in online repositories. The names of the repository/repositories and accession number(s) can be found at: https://osf.io/sehnf/.

## Ethics statement

The studies involving human participants were reviewed and approved by Ethikkommission der Deutschen Gesellschaft für Sprachwissenschaft (Ethics Committee of the German Linguistics Association). The patients/participants provided their written informed consent to participate in this study.

## Author contributions

OÖ, IS, and NG contributed to conception and design of the study. OÖ and BÇ organized the database. OÖ performed the statistical analysis. OÖ, IS, BÇ, and ZÖ wrote the first draft of the manuscript. OÖ, IS, BÇ, ZÖ, and NG provided feedback on further versions and wrote sections of the manuscript. All authors contributed to manuscript revision, read, and approved the submitted version.

## Footnotes

^1^The testing occurred between March and June 2022 which was a time-period when COVID-regulations were still in place and special hygiene and mask regulations were part as part of the laboratory testing protocols.

^2^We thank Yagmur Baydar for recording and sharing the audio files with us.

## Appendix A

### 1. Divergence point estimation

In Figure 2, the (uncorrected) divergence point estimates are represented by the purple triangle-shaped symbols in each panel. Recall that the NP2 onset began at 2,300 ms in all experiments. The divergence point in Experiment 1 (monolingual speakers, webcam-based eye-tracking) fell within the region of interest for the predictive effect of the case, at 2,000 ms (*z* = 2.77, *p* = 0.00554). In contrast, in Experiment 3 (HSs, in-lab eye-tracking), the divergence point was outside of the region of interest, at 2,600 ms (*z* = 2.56, *p* = 0.0106). There was no divergence point in Experiment 2 (HSs, webcam-based eye-tracking) at all in the region of interest. Thus, at the group level, we locate the effect of predictive processing of case-marking cues in the monolingual speakers, but not in the HSs, regardless of the eye-tracking set-up.

It is important to consider the corrected multiple comparisons which is why we have calculated also the Bonferroni-corrected and FDR-controlled divergence point estimates (Stone et al., 2021). The aim here is to limit the rate of false positives (Type I error) which might arise given the big number of time-points that are statistically compared to each other using this method. However, none of the estimated divergence points in our analysis survived the correction which is why we do not report them here. Plausible explanations for this are the low data resolution from webcam-based eye-tracking in Experiment 1 and 2 and the limited sample size in Experiments 2 and 3.
